# Anti-platelet therapy holds promises in treating adenomyosis: experimental evidence

**DOI:** 10.1186/s12958-016-0198-1

**Published:** 2016-10-10

**Authors:** Bo Zhu, Yumei Chen, Xiaolu Shen, Xishi Liu, Sun-Wei Guo

**Affiliations:** 1 Department of Obstetrics and Gynecology, The People’s Hospital, Wenzhou, Zhejiang 325800 China; 2 Shanghai Key Laboratory of Female Reproductive Endocrine-Related Diseases, Shanghai, 200011 China; 3 Shanghai Obstetrics and Gynecology Hospital, Fudan University, 419 Fangxie Road, Shanghai, 200011 China

**Keywords:** Adenomyosis, Generalized hyperalgesia, Hotplate latency, Mouse, Ozagrel, Platelet, Uterine contractility

## Abstract

**Background:**

Recently emerging evidence indicates that endometriotic lesions are wounds undergoing repeated tissue injury and repair (ReTIAR), and platelets induce epithelial-mesenchymal transition (EMT), fibroblast-to-myofibroblast transdifferentiation (FMT), leading ultimately to fibrosis. Due to the commonality of cyclic bleeding as in endometriosis, adenomyotic lesions are also wounds that undergo ReTIAR, and we have recently provided evidence corroborating platelet-induced EMT, FMT and fibrogenesis in adenomyosis. This study sought to evaluate the effect of antiplatelet therapy in a mouse model of adenomyosis.

**Methods:**

Adenomyosis was induced in 57 female ICR mice with neonatal dosing of tamoxifen, while another 12 (group C) were dosed with solvent only, serving as a blank control. Starting from 4 weeks after birth, hotplate test was administrated to all mice every 4 weeks. At the 16th week, all mice with induced adenomyosis were randomly divided into 6 groups: untreated, low- and high-dose Ozagrel, low- and high-dose anti-mouse GPIbα polyclonal IgG antibody to deplete platelets, and isotype-matched inert IgG non-immune antibody. Group C received no treatment. After 3 weeks of treatment, they were hotplate tested again, their uterine horns and brains were harvested, and a blood sample was taken to measure the plasma corticosterone level by ELISA. The left uterine horn was used for immunohistochemistry analysis. The brainstem nucleus raphe magnus (NRM) sections were subjected to immunofluorescence staining for GAD65. The depth of myometrial infiltration and uterine contractility were evaluated.

**Results:**

We found that both Ozagrel treatment and platelet depletion dose-dependently suppressed myometrial infiltration, improved generalized hyperalgesia, reduced uterine contractility, and lowered plasma corticosterone levels, improved the expression of some proteins known to be involved in adenomyosis and slowed down the process of fibrogenesis. It also elevated the number of GAD65-expressing neurons in the brainstem NRM, possibly boosting the GABAergic inhibition of pain due to adenomyosis.

**Conclusion:**

This study further provides evidence that platelets play important roles in the development of adenomyosis. Anti-platelet treatment is efficacious in suppression of myometrial infiltration, improving generalized hyperalgesia, reducing uterine hyperactivity and systemic corticosterone levels. Collectively, these results demonstrate that anti-platelet therapy seems to be promising for treating adenomyosis.

**Electronic supplementary material:**

The online version of this article (doi:10.1186/s12958-016-0198-1) contains supplementary material, which is available to authorized users.

## Background

Adenomyosis is a common gynecologic disorder with a poorly understood pathogenesis [1]. As in endometriosis, it is characterized by the ectopic deposition and growth of endometrial glands and stroma deep and haphazardly into the myometrium [1]. It shares with endometriosis many similarities in terms of estrogen-dependency, progesterone resistance, symptomology, and many molecular aberrations but differs in risk factors, age at onset and, possibly, etiology [2]. Similar to endometriosis, our current knowledge of its pathophysiology is still woefully inadequate. Consequently, treatment of adenomyosis has been a challenge [3], with hysterectomy being the treatment of choice for severe symptomatic adenomyosis. Thus, medical treatment of adenomyosisis still an unmet medical need.

Adenomyosis is first and foremost viewed as an estrogen-dependent disease, featuring increased local production of estrogen [4]. It also displays signs of inflammation, characterized by the constitutive activation of NF-kB [5], increased macrophage infiltration [6], and elevated expression of COX-2, a rate-limiting enzyme in catalyzing prostaglandin (PG) E2 (PGE_2_) [7], and increased production of proinflammatory cytokines and chemokines [8]. All existing therapeutics for adenomyosis are hormonal drugs.

As with endometriosis, the ectopic endometrium in adenomyosis also experience cyclic bleeding. Yet bleeding, an indication of vascular injury, is a cardinal hallmark of a wound or tissue damage. Consequently, a physiological process, called wound healing or tissue repair, ensues. As such, platelets must be involved, as shown recently for endometriosis [9]. In fact, based on serial immunohistochemistry analyses of ectopic endometrium in a mouse model of adenomyosis, we recently report that activated platelets coincide with TGF-β1 release and the induction of TGF-β/Smad signaling pathway in adenomyosis, as well as evidence of epithelial-mesenchymal transition (EMT) and fibroblast-to-myofibroblast transdifferentiation (FMT), resulting ultimately in fibrosis [10] and also smooth muscle metaplasia (Shen et al., unpublished data). These observations are confirmed in human adenomyosis [11]. Therefore, due to the commonality shared with endometriosis, i.e., cyclic bleeding, adenomyotic lesions behave just like endometriotic lesions, which are essentially wounds that undergo repeated tissue injury and repair (ReTIAR) [9, 12, 13].

In light of the important roles that platelets play in the development of endometriosis [9, 14, 15] and adenomyosis [10, 11], one may wonder as whether anti-platelet therapy may have any potential in treating adenomyosis. This study was undertaken to test the hypothesis that anti-platelet treatment, by either platelet depletion or administration of Ozagrel, indeed has potential for therapeutic purposes.

## Methods

### Chemicals

Ozagrel, a thrmboxane A2 (TXA_2_) synthese inhibitor [16], was purchased from YaoDa Pharmacology Industry Company (Shenyang, China) and was dissolved in 0.9 % normal saline for intraperitoneal administration. The rat anti-mouse GPIbα polyclonal IgG antibody and its isotype-matched non-immune rat anti-mouse IgG antibody were purchased from Emfret Analytics (Eibelstadt, Germany). Tamoxifen citrate was purchased from Fudan Forward Pharmaceutical Company (Shanghai, China). All other chemicals were purchased from Sigma unless stated otherwise.

### Animals and the procedure for induction of adenomyosis

Four pregnant ICR mice with a gestational age of 15–16 days were purchased from Shanghai Laboratory Animal Corporation (Shanghai, China) and each of them was housed in a single cage during the rest of the gestation period and the ensuing birth and nursing period. Their pups (1 day after birth) were sexed and the female pups were selected for use in this study. The same litter of pups and the dam were housed in the same cage until weaned. All mice were housed in an animal care facility under controlled conditions (20 °C, 12:12 light/dark cycle with lights on at 6:00 AM) and had free access to chow and fresh water.

Following Parrott et al. [17, 18], and as reported previously [2, 19, 20], adenomyosis was induced by orally dosing female neonatal mice with 1 mg/kg tamoxifen suspended in peanut oil/lecithin/condensed milk mixture (2:0.2:3, by volume) at a dose volume of 5 μl/g bodyweight from day 2 to day 5 after birth. Female control neonatal mice, selected randomly, were fed similarly with the same amount of solvent, without tamoxifen. When these female mice reached 3 weeks of age, they were weaned and separated from the dams.

All experiments were performed under the guidelines of the National Research Council’s *Guide for the Care and Use of Laboratory Animals* [21] and approved by the institutional experimental animals review board of Shanghai OB/GYN Hospital, Fudan University.

### Experimental protocol

This experiment was conducted side-by-side with another experiment evaluating the efficacy of epigallocatechin-3-gallate (EGCG) in treating adenomyosis in mice, as reported in [20]. Fifty-six female neonatal pups were orally dosed with tamoxifen from day 2 to day 5 after birth, while another 12 were dosed in similar fashion with the solvent only (control group, or group C). Starting from 4 weeks after birth, hotplate test was administered to all mice every 4 weeks, as described previously [2, 19] (see Additional file 1 for full description). At the 16th week after birth, all mice dosed with tamoxifen were randomly divided into6 groups of roughly equal size, Group U (*n* = 9), or the untreated group, received the vehicle only. Another two groups received, for 3 weeks, daily intraperitoneal (i.p.) administration of either low-dose (12.5 μg/g) Ozagrel (Group L, *n* = 10) or of high-dose (25 μg/g) Ozagrel (Group H, *n* = 10). The Ozagrel doses were determined based on the dosage given to an adult female and then converted from human to mouse based on body surface area and further adjusted based on each mouse’s bodyweight measured every day before Ozagrel administration. The mice in group C received no treatment at all and served as a blank control. The other three groups were intravenously (i.v.) administrated with different doses of rat anti-mouse GPIbα polyclonal IgG and non-immune rat anti-mouse IgG: Group LD (*n* = 10) received a low-dose (1 μg/g) rat anti-mouse GPIbα polyclonal IgG treatment; Group HD (*n* = 9) received a high-dose (2 μg/g) rat anti-mouse GPIbα polyclonal IgG treatment; Group NI (*n* = 8) received 1 μg/g non-immune (NI) rat anti-mouse IgG isotope-matched with the anti-GPIbα antibody; The mice in group C were treated the same as above. The dosages of rat anti-mouse GPIbα polyclonal IgG were determined based on instructions provided by Emfret Analytics.

**Fig. 1:**
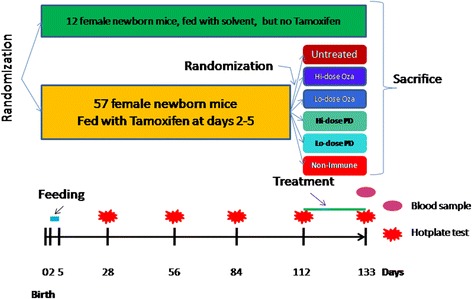
Schematic illustration of the experiment design of this study. Untreated: mice that received no treatment; Lo-dose Ozagrel (Oza): mice treated with 12.5 μg/g Oza (low-dose); Hi-dose Oza: mice treated with 25 μg/g Oza (high-dose). Lo-dose PD: mice treated with 1 μg/g rat anti-mouse GPIbα polyclonal IgG antibody for platelet depletion (PD); Hi-dose PD: mice treated with 2 μg/g rat anti-mouse GPIbα polyclonal IgG antibody; Non-immune: received 1 μg/g non-immune (NI) rat anti-mouse IgG isotope-matched with the anti-GPIbα antibody See text on experiment protocol for more details

After the 3-week-long treatment period (at the 19th week), the final hotplate test was administered to all the mice with or without induced adenomyosis, after bodyweight measurement. A 0.5 ml blood sample was taken between 9:00 and 15:00 of the dayfrom each mouse, and was used for the measurement of plasma corticosterone levels by enzyme linked immunosorbent assay (ELISA, see Additional file 1 for more details). After the blood samples were taken, all mice were sacrificed by perfusing the heart with formalin. For each mouse, both uterine horns were harvested and the uterine weight was recorded. The left uterine horn was used for uterine contractility measurement (described in Additional file 1), and the right one was fixed in 4 % paraformaldehyde immediately after collection and then embedded in paraffin. The brains of all mice were harvested and analyzed (described below). The experiment design is shown schematically in Fig. 1.

We evaluated the depth of myometrial infiltration of ectopic endometrium following the criteria of Bird et al. [22], as reported previously [2]. Briefly, Grade 1 was defined to be the case where penetration of the ectopic endometrium into superficial myometrium; Grade 2, penetration into mid-myometrium; and Grade 3, penetration beyond mid-myometrium. For ease of statistical analysis, Grade 0 was recorded when there was a complete absence of any ectopic endometrium in the myometrium.

For histological examination, serial 4-μm sections were obtained from each paraffin-embedded tissue block, and then 3 randomly selected sections were chosen for H&E staining to confirm pathologic diagnosis, as described previously [2, 19]. If endometrial glands and stroma were seen to be infiltrated into in myometrium, the diagnosis of adenomyosis was made.

### Histochemistry and immunohistochemistry analyses

As described above, the right horn of uterus was fixed in 4 % formalin, and then embedded in paraffin. From each tissue block, serial 4-μm sections were obtained and subjected to H&E staining to confirm pathologic diagnosis of adenomyosis, which was characterized by the presence of endometrial glands and stroma that are completely enveloped by myometrium and discontinuous with the endometrial cavity [23, 24].

The rabbit polyclonal antibodies against progesterone receptor isoform B (PR-B, ab2765; Abcam, Hong Kong, China), phosphorylated-p65 (ab30623; Abcam), cyclooxygenase 2 (COX-2, #4842; CST, Boston, USA), oxytocin receptor (OTR, ab115664; Abcam), transient receptor potential cation channel, subfamily V, member 1 (TRPV1, ab31895;Abcam), collagen I (ab292; Abcam), collagen IV (ab6586; Abcam), and the rat monoclonal antibody against F4/80 (MCA497G; AbDSerotec, Cambridge, England), CD41(ab33661), diluted to1:50,1:80,1:200, 1:100, 1:1000,1:100, 1:500, 1:200 and 1:100, respectively, were used as primary antibodies.

Serial 4-μm sections were made from paraffin-embedded tissue blocks. After routine deparaffinization and dehydration, some sections were randomly selected to be heat-retrieved with Tis-EDTA buffer (0.5 mol/L PH 9.0) over 98 °C for a total of 30 min for immunostaining for PR-B, p-p65, COX-2, OTR and TRPV1, and the others were heat-retrieved with citric acid (0.01 mol/L pH 6.0) over 98 °C for a total of 30 min for immunohistochemistry analysis of collagen I, collagen IV, and F4/80. Then all sections were cooled naturally at room temperature, and then incubated with the primary antibodies at 4 °C overnight. After the sections were rinsed with PBS, they were incubated with the secondary antibody (Sunpoly-HII, BioSunTechnclogy, Shanghai, China) for half an hour, or, for F4/80, with the goat anti-rat antibody (AbDSerotec) for 1 hour. The bound antibody complexes were stained for 3–5 minor until appropriate for microscopic examination with diaminobenzidine (DAB) (BioSunTechnclogy Co., Ltd) and then counterstained with hematoxylin and mounted.

Images were obtained with the microscope (Olympus BX51, Olympus, Tokyo, Japan) fitted with a digital camera (Olympus DP70, Olympus). Five randomly selected images from 10 to 12 images on 2–4 sections of each mouse were taken for each immunostaining marker to obtain a mean optional density value by Image Pro-Plus 6.0 (Media Cybernetics, Inc., Bethesda, MD, USA) as reported in [25]. Staining was defined via color intensity, and a color mask was made. The mask was then applied equally to all images, and measurement readings were obtained. Immunohistochemical parameters assessed in the area detected included (a) integrated optical density (IOD); (b) total stained area (S); and (c) mean optical density (MOD), which is defined as MOD = IOD/S, equivalent to the mean intensity of stain in all positive cells.

For F4/80, we counted the number of F4/80-positive macrophages from five randomly selected images and calculated their average. Myometrial OTR staining scores were calculated by multiplying the percentage of positive OTR cells per section (0–100 %) by a semi-quantitative classifier for immunohistochemical staining intensity, which was scored as 0 if there was a complete absence of any staining, 1 for weak staining, 2 for moderate staining, and 3 for strong staining. Consequently, the staining scores ranged from a minimum of 0 to a maximum of 300. We used the mean score averaged over 5 randomly selected images.

For all markers, the staining levels were scored on ectopic endometrium in mice with induced adenomyosis. For mice in the control group or in mice without ectopic endometrium due to treatment, they were scored in the endometrium. We counted the number of macrophages (F4/80-positive) infiltrated into the ectopic endometrium in the entire focal field for mice with adenomyosis. For the mice in control group or mice that had none such lesion due to treatment, the number of F4/80-positive macrophages in endometrium was counted.

The mouse spleen tissue was used for the positive immunostaining of macrophages, while breast cancer tissue sections were used for positive control for other markers, Negative control sections were processed similarly, but using a non-immune rabbit or rat IgG instead of the primary antibody, or by omitting the primary antibody from the incubation medium. No positive reaction was observed under these conditions. All sections were inspected a single investigator (BZ) without the knowledge of the group identity. The positive and negative controls are shown in Additional file 1: Figure S2 of Supplemental Information.

### GAD65 immunofluorescence staining of neurons in brainstem nucleus raphe magnus

The procedure has been reported previously in [26]. Briefly, the mouse brains containing the nucleus raphe magnus (NRM) were harvested and immediately embedded in O.C. T. compound in liquid phlegm after the mice were sacrificed. The NRM sections were between 5.68 and 6.48 mm to the bregma and the NRM was located 1.72–2.68 mm interaurally, as described previously [26]. Serial 6-μm sections were performed on a cryostat for each block and stored at the temperature below −20 °C until use. The sections were incubated in goat anti mouse serum (BioSunTechnclogy) for 10 min and then incubated in mouse anti primary antibody against GAD65 (ab26113, Abcam; 1:1,000) at 4 °C overnight. GAD65 is expressed in the cytoplasm of presynaptic neurons. The sections were rinsed with PBS (pH 7.4) and incubated in secondary antibody mixed with DyLight649 (E032610, Earthox, San Francisco, USA) for one hour, and then rinsed with PBS (pH 7.4) again and mounted.

Images were obtained with a microscope (Olympus BX51) fitted with a digital camera (Olympus DP70). Five randomly selected images out of 6–7 sections of each mouse were taken for each immunostained parameter to count the numbers of GAD65-positive (red) cells in the NRM, located between 1.72 mm and 2.68 mm interaurally, and the mean was calculated.

### Statistical analysis

Comparison of the distributions among two or more groups of continuous variables was made using the Wilcoxon and Kruskal-Wallis tests, respectively, and the paired Wilcoxon test was used when the before-after comparison was made for the same group of subjects. Pearson’s or Spearman’s rank correlation coefficient was used when evaluating correlations between two variables when both variables were continuous or when at least one variable was ordinal. To see whether Ozagrel treatment or platelet depletion and other possible factors were responsible for the change in hotplate latency before and after the treatment, a multiple linear regression model was used. To see whether there is trend in immunostaining levels as a function of the depth of myometrial infiltration, Jonckheere trend was used.

To determine correlates of depth of myometrial infiltration, we used the Cox proportional odds logistic regression model. This model assumes, implicitly, that the data were ordered categorical data, with an implicit underlying order (scale of severity) in the data [27], with 4 categories corresponding to Grade 0, I, II, and III infiltration.

*P* values of less than 0.05 were considered statistically significant. All computations were made with R statistics software system version 3.3.1 [28].

## Results

Consistent with Parrott et al. [17, 18] and as previously reported [2, 19], we found that adenomyosis was successfully induced in all (100 %) mice dosed with tamoxifen but none in un-dosed mice.

Ozagrel was well-tolerated, as no mice in either LO or HO group died, and we found nothing unusual in these mice. In HD group, however, 1 mouse died after it received the 4th injection of the depletion antibody, and 2 appeared to be lethargic. In the LD group, no mice died and nothing appeared unusual. There was no difference in platelet counts between the mice in groups UT, NI, LO, and HO at the end of the experiment. However, the platelet count in mice in both LD and HD groups was reduced by 99.6 and 99.7 % as compared with those in the NI group, demonstrating the effectiveness of platelet depletion in these two groups.

### Treatment effect on the depth of myometrial infiltration, hotplate latency, and uterine and bodyweight

**Fig. 2:**
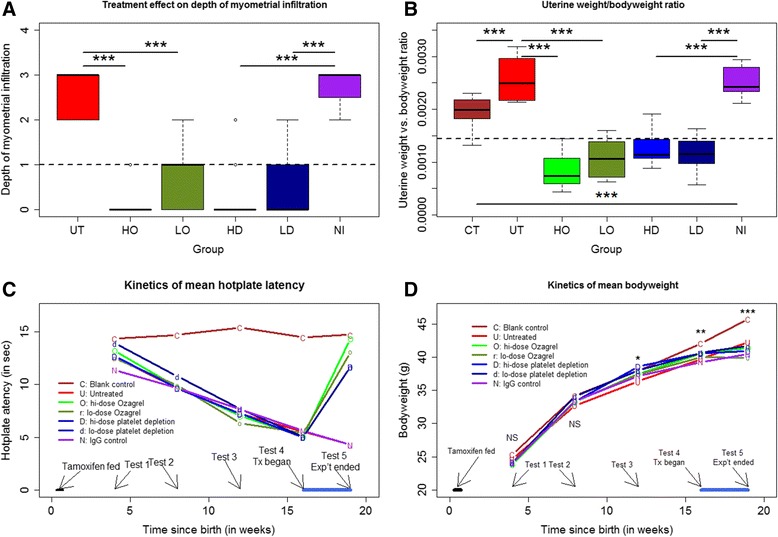
Some summary results of the experiment. **a** Boxplot of the depth of myometrial infiltration among different groups of mice with induced adenomyosis. **b** Boxplot of uterine vs. body weight ratio at the end of 3-week-long RSV treatment among different groups of mice. **c** Kinetic changes in average hotplate latency among different treatment groups. **d** Kinetics of mean bodyweight among different treatment groups. UT: Untreated group; CT: Blank control group; LO: low-dose Ozagrel group; HO: high-dose Ozagrel group; LD: platelet depletion using low-dose antibody; HD: platelet depletion group using high-dose antibody; NI: mock depletion using non-immune antibody; Tx: Treatment; Exp’t: Experiment. The arrows showing different tests are the administrated hotplate tests. The *blue line* in (**c**) and (**d**) indicates the duration of the treatment. In (**a**) and (**b**), the statistical significance of the difference between the testing group and the comparison group was indicated, and “***” means that the *p*-value is less than 0.001. In (**d**), the statistical significance was referring to the difference among the 7 groups of mice. *: *p* < 0.05; **: *p* < 0.01; ***: *p* < 0.001

We first evaluated the effect of Ozagrel treatment or platelet depletion on the depth of myometrial infiltration. We found that, compared with untreated mice, mice treated with either low- or high-dose Ozagrel had significantly less infiltration (both *p*-values <0.001; Fig. 2a). Compared with NI mice, mice in either LD or HD group also had significantly less infiltration (both *p*-values <0.001; Fig. 2a). Mice in HO and HD groups appeared to have less infiltration than those in the LO or LD group (Fig. 2a).

The multiple linear regression analysis suggested that both Ozagrel treatment and platelet depletion significantly and dose-dependently reduced the depth of myometrial infiltration (regression coefficient β = −0.956, *p* = 3.2×10^−7^, and β = −0.627, *p* = 1.5×10^-6,^ respectively, *R* ^*2*^ = 0.62), but NI mice had deeper infiltration (β = 0.856, *p* = 0.015). The Cox regression analysis yielded similar results (all three *p*-values <0.028).

We found that there is a significant difference in uterine weight vs. bodyweight ratio among the 7 groups of mice (*p* < 0.001; Fig. 2b). Using the ratio as a dependent variable and the bodyweight after treatment, the induction of adenomyosis, dose of Ozagrel, the non-immune IgG injection or not, and the dose of antibody used in platelet depletion as covariates, we found, via a linear multiple regression, that the non-immune IgG was positively associated with the ratio (*p* < 0.01; *R* ^*2*^ = 0.60; Fig. 2b) while both Ozagrel and anti-platelet doses were negatively associated with the ratio (both *p*-values <0.001). Similar results were obtained for the uterine weight (data not shown).

The induction of adenomyosis was significantly associated with reduced latency just 4 weeks after birth or 23 days after the completion of tamoxifen dosing (*p* < 0.05; Fig. 2c). At 8 weeks after birth, the difference in hotplate latency amount the four groups of mice became very pronounced (*p* < 0.001; Fig. 2c), with the mice with induced adenomyosis all having reduced latency (*p* < 0.001). At 12 and 16 weeks after birth, the mice with induced adenomyosis had further progressively reduced hotplate latency (both *p*-values <0.001; Fig. 2c). In all mice with induced adenomyosis, the latency evaluated at week 8, 12 and 16 was all significantly decreased as compared with the previously evaluated latency (all *p*-values <0.001; Fig. 2c).

After platelet depletion or treatment with Ozagrel for 3 weeks, however, the hotplate latency was significantly improved in a dose-dependent fashion (β = 4.008, *p* < 0.001, and β = 1.792, *p* < 0.001, respectively, in a multiple linear regression analysis, *R* ^*2*^ = 0.73; Fig. 2c). In contrast, the presence of adenomyosis and the injection of the dummy antibody were associated with decrease in hotplate latency (*p* < 0.001 and *p* < 0.01, respectively).

While there was no difference in bodyweight among the 7 groups of mice at 4 and 8 weeks after birth (both *p*-values >0.05; Fig. 2d), the difference became statistically and progressive significant starting from week 12 (*p* < 0.05 and *p* < 0.01), and was significant at the end of the experiment (*p* < 0.001; Fig. 2d). The multiple linear regression analysis using pre-treatment bodyweight, presence of adenomyosis, and the dose of Ozagrel, the non-immune IgG injection or not, and the dose of antibody used in platelet depletion as covariates, we found that only the induction of adenomyosis was negatively associated with the bodyweight (*p* < 0.001; *R* ^*2*^ = 0.66) while the pre-treatment bodyweight was positively associated with the bodyweight (*p* < 0.001). In other words, neither Ozagrel treatment nor platelet depletion had any impact on bodyweight, but the induction of adenomyosis had a negative impact due, possibly, to adenomyosis-associated pain and/or pain-induced suppression of appetite.

### Treatment effect on uterine contractility

**Fig. 3:**
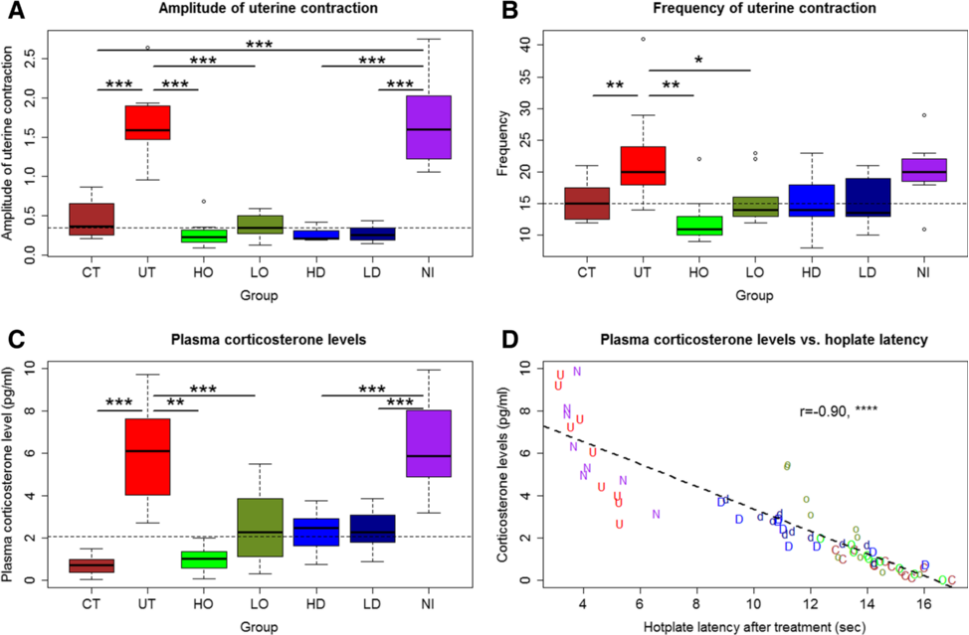
Summary results on uterine contractility and plasma corticosterone levels. **a** Boxplot of the amplitude of uterine contractility among different groups of mice with induced adenomyosis. **b** Boxplot of the frequency of uterine contractility among different groups of mice. **c** Boxplot of the plasma corticosterone levels among different groups of mice. **d** Scatter plot showing the relationship between hotplate latency and the plasma corticosterone levels. In (**a**), (**b**) and (**c**), the statistical significance of the difference between the testing group and the comparison group was indicated. *: *p* < 0.05; **: *p* < 0.01; ***: *p* < 0.001. In (**d**), each letter represents one mouse, and the alphabet indicates the group identity, which is the same as used in Fig. 2c, d. The correlation coefficient, with its statistical significance level, is shown in the figure

There was a significant difference in the amplitude of uterine contractility after drug treatment among the 7 groups (*p* < 0.001; Fig. 3a). In particular, both untreated and NI mice had a significantly higher amplitude as compared with the mice without adenomyosis (both *p*-values < 0.001; Fig. 3A). Regressing the amplitude on the Ozagrel dose, the presence of adenomyosis, the non-immune IgG injection or not, and the dose of antibody used in platelet depletion indicated that, while Ozagrel treatment and platelet depletion were both negatively associated with the amplitude in a dose-dependent fashion (both *p*-values < 0.001), the induction of adenomyosis and the IgG injection were positively and significantly associated with increased amplitude (*p* < 0.001, and *p* < 0.01, respectively; *R* ^*2*^ = 0.60).

Similarly, there was a significant difference in the frequency of uterine contractility after drug treatment (*p* < 0.01; Fig. 3B). Regressing the frequency (log-transformed to enhance normality) on the uterine weight vs. bodyweight ratio, Ozagrel dose, the presence of adenomyosis, the IgG injection or not, and the dose of antibody used in platelet depletion indicated that the induction of adenomyosis was positively associated with the contractile frequency (*p* < 0.01; *R* ^*2*^ = 0.30) while Ozagrel treatment and platelet depletion were both negatively associated with the frequency (*p* < 0.001 and *p* < 0.01, respectively).

The contractile amplitude correlated positively with the contractile frequency (*r* = 0.51, *p* < 0.001). Both the amplitude and frequency were found to correlate positively with the uterine vs. bodyweight ratio (*r* = 0.79, *p* < 0.001, and *r* = 0.52, *p* < 0.001).

### Treatment effect on plasma level of CORT

We found that there is a significant difference in plasma CORT levels among the 7 groups of mice (*p* < 0.001; Fig. 3c). In particular, the untreated mice had a significantly elevated CORT levels as compared with mice without adenomyosis, so did the NI mice (both *p*-values <0.001; Fig. 3c). Regressing the plasma CORT level (log-transformed to enhance normality) on the Ozagrel dose, the presence of adenomyosis, the non-immune IgG injection or not, and the dose of antibody used in platelet depletion indicated that both the induction of adenomyosis and the injection of the dummy antibody were positively associated with the CORT levels (*p* < 0.001 and *p* < 0.05, respectively) while both Ozagrel treatment and platelet depletion were dose-dependently and negatively associated with the CORT levels (both *p*-values <0.001; *R* ^*2*^ = 0.59). We also found that the CORT levels correlated negatively with the hotplate latency (*r* = −0.90, *p* < 0.001; Fig. 3d), suggesting that pain severity may be positively associated with the severity of stress.

### Effect of antiplatelet treatment on platelet aggregation, macrophage infiltration, and select markers in ectopic endometrium

**Fig. 4:**
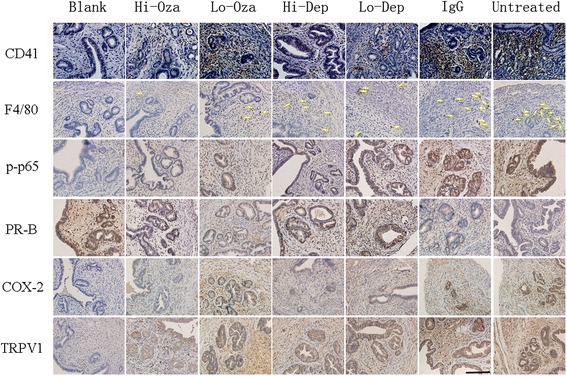
Representative photomicrographs of indicated immunostaining in eutopic (for blank control group) or ectopic (all other groups) endometrium among different treatment groups, which are arranged in different columns. CD41: CD41-labeled platelets; F4/80: F4/80-labled macrophages (indicated by yellow arrows); p-p65: phosphorylated form of NF-kB p65 subunit; PR-B: progesterone receptor isoform B; COX-2: Cyclooxygenase 2; TRPV1: transient receptor potential cation channel, subfamily V, member 1. Blank: blank control endometrium; Hi-Oza: high-dose Ozagrel group; Lo-Oza: low-dose Ozagrel group; Hi-Dep: platelet depletion with high-dose antibody; Lo-Dep: platelet depletion with low-dose antibody; IgG: non-immune mock antibody. Scale bar = 125 μm

**Fig. 5:**
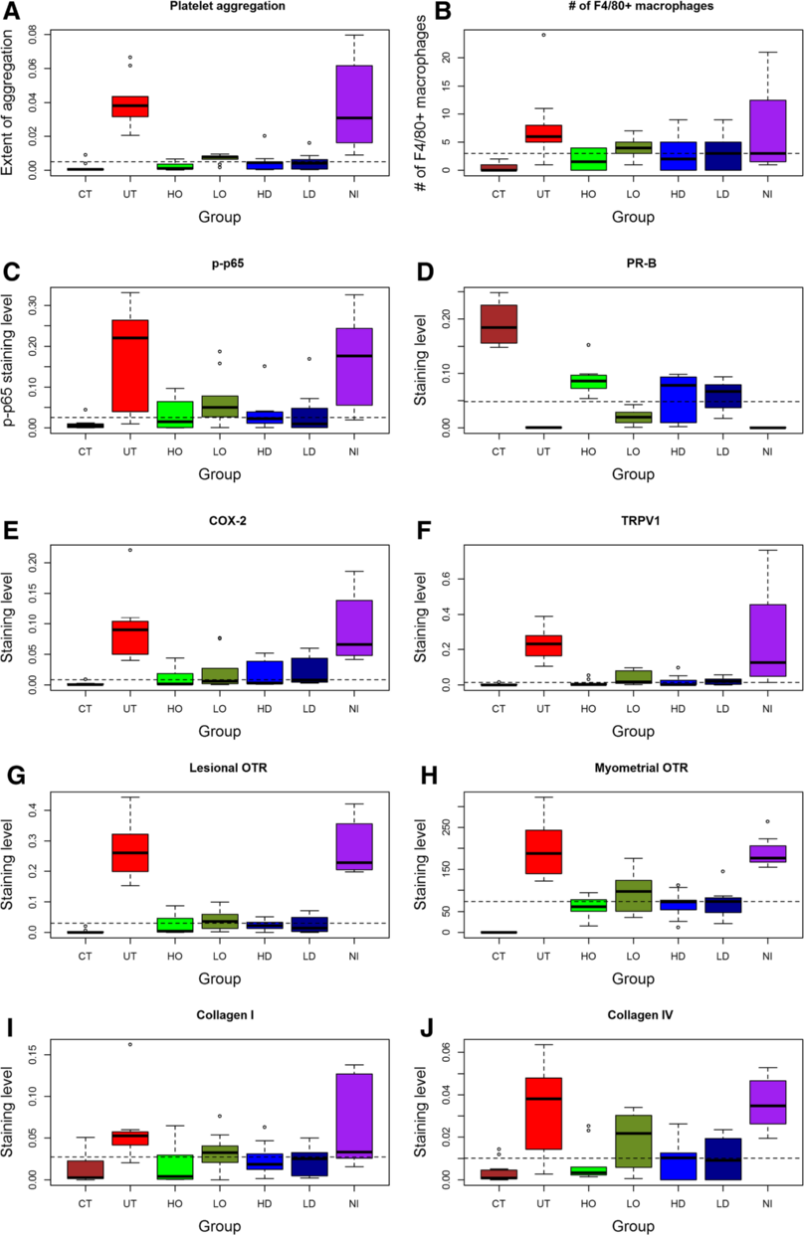
Summary of immunohistochemistry results. Boxplot of immunoreactivity against CD41 (**a**), the number of F4/80+ positive macrophages (**b**), p-p65 **c**, PR-B (**d**), COX-2 (**e**), TRPV1 (**f**),OTR (**g**), myometrial OTR (**h**), Collagen I (**i**), and Collagen IV (**j**) in ectopic/eutopic endometrium. The group labels are the same as used in Fig. 2

**Fig. 6:**
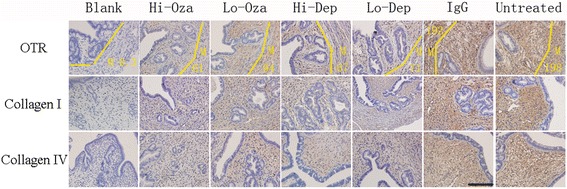
Representative immunohistochemisty staining of markers of smooth muscle metaplasia and fibrosis in ectopic and eutopic endometrium. Different rows indicate different proteins in different groups (arranged in different columns) with different doses of Ozagrel, different doses of rat anti-mouse GPIbα polyclonal IgG and non-immune rat anti-mouse IgG. For oxytocin receptor (OTR), M stands for staining in the myometrium, stromal and gland epithelium was separately evaluated. Magnification in all figures: ×400. Scale bar = 125 μm

We evaluated the immunoreactivity results for all mice. Figure 4 shows the extent of platelet aggregation and of macrophage infiltration and p-p65, PR-B, COX-2, and TRPV1 immunostaining in ectopic endometrium among different groups. For COX-2, the staining was predominantly localized in the cytoplasm of glandular epithelial cells in ectopic and eutopic endometrium. Both PR-B and p-p65 staining was localized primarily in the nuclei of glandular epithelial cells of eutopic and ectopic endometrium while TRPV1 staining was seen mainly in the cytoplasm and cell membranes of glandular epithelial cells;

We found that there was a significant difference in immunoreactivity to PR-B, p-p65, COX-2, and TRPV1 in ectopic/eutopic endometrium and to OTR in myometrium among different groups (all *p*-values <0.01; Figs. 5 and 6, and Table 1). In particular, multiple linear regression analyses (all immunoreactivity levels were square-root transformed to improve normality unless stated otherwise) indicated that while adenomyosis induction was associated with the increase (decrease for PR-B) while Ozagrel treatment or platelet depletion was associated, in a dose-dependent manner, with a significant reduction (increase for PR-B) of immunoreactivity to all these proteins or the extent of platelet aggregation/macrophage infiltration (all *p*-values <0.01, with *R* ^*2*^ ranging from 0.31 to 0.76; Table 1 and Fig. 5).

**Table 1:** Results from early/later platelet depletion experiment. All results were based on multiple regression analyses with the independent variable square-root transformed and dummy variables indicating the presence or absence of adenomyosis, non-immune IgG antibody injection or not, the dosage of antibody to deplete platelets and the dosage of Ozagrel as co-variables

Name	Induction of adenomyosis	Ozagrel treatment	Platelet depletion	*R* ^*2*^
Extent of CD41+ platelet aggregation	↑ ***	↓ ***	↓ ***	0.54
Number of infiltrated F4/80+ macrophages	↑ ***	↓ **	↓ *	0.30
Phosph-p65	↑ ***	↓ **	↓ **	0.31
PR-B	↓ ***	↑ ***	↑ ***	0.73
COX-2	↑ ***	↓ ***	↓ ***	0.48
TRPV1	↑ ***	↓ ***	↓ ***	0.47
OTR	↑ ***	↓ ***	↓ ***	0.58
Collagen I	↑ ***	↓ ***	↓ *	0.29
Collagen IV	↑ ***	↓ **	↓ ***	0.33
Myometrial OTR	↑ ***	↓ ***	↓ ***	0.77

↓: Denotes that the immunoreactivity to this protein at hand was significantly decreased based on multiple linear regression analysis;↑Denotes that the immunoreactivity to this protein of interest was significantly increased based on multiple linear regression analysis. The *R* ^*2*^ value of the corresponding regression model is shown at the right-most column. Symbols of statistical significance levels: *: p<0.05; **: p<0.01; ***: p<0.001

In addition to these markers, we also performed immunostaining of lesional OTR, a marker of SMM, and collagen I and IV, markers of fibrosis, in adenomyotic lesions, as well as OTR in myometrium, which was likely responsible for uterine hyperactivity. OTR staining was localized in both the cell membrane and the cytoplasm of glandular epithelial and stromal cells as well as myometrial smooth muscle cells (Fig. 6). We scored OTR staining levels in epithelial/stromal cells and myometrial muscle cells separately. No difference in OTR staining levels in the epithelial component was found (data not shown), and hence only the data in the stromal component were demonstrated. Both collagen I and collagen IV staining was seen nearly uniformly in extracellular matrix of the ectopic endometrial stromal tissues, irrespective of the proximity to the glandular epithelial cells or not.

We found that for all these markers, the presence of adenomyosis and, in the case of lesional and myometrial OTR staining, the injection of non-immune antibodies were positively associated with the staining levels while Ozagrel treatment and platelet depletion were associated, in a dose-dependent manner, with a significant reduction of immunoreactivity to all these proteins (all *p*-values <0.001; Fig. 5; Table 1).

We found that the immunostaining levels of these proteins were all highly correlated, with the positive correlation coefficients ranged from 0.69 to 0.95 (PR-B excluded; all *p*-values <0.001) and the negative correlation coefficients ranged from −0.72 to −0.93 (all *p*-values < 0.001 for PR-B vs. others).

**Fig. 7:**
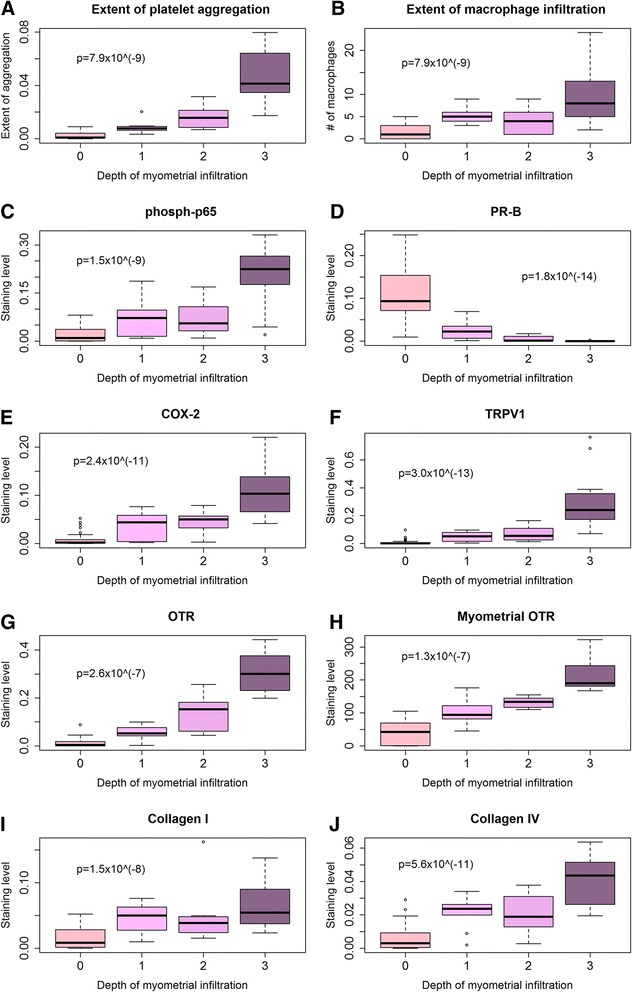
Summary results on immunohistochemistry measures as a function of the depth of myometrial infiltration. Boxplot of the extent of platelet aggregation (**a**), extent of macrophage infiltration (**b**), immunoreactivity against p-p65 (**c**), PR-B (**d**), COX-2 (**e**), TRPV1 (**f**), OTR (**g**), myometrial OTR (**h**), collagen I (**i**), and collagen IV (**j**) in ectopic endometrium as a function of the depth of myometrial infiltration of endometrial tissues. The *p*-value shown in each figure is the statistical significance of the Jonckheere trend test

We found that the extent of platelet aggregation and of the macrophage infiltration as well as the immunoreactivity to PR-B, *p*-p65, COX-2 and TRPV1 were all highly correlated with the depth of myometrial infiltration (all positive except PR-B, which was negative, and the Spearman’s correlation coefficients ranged from 0.69 to 0.88 (−0.87 for PR-B), all *p*-values <0.001). The Jonckheere trend test indicated that all these immunostaining levels were significantly associated with the depth of myometrial infiltration (all *p*-values <0.001; Fig. 7). A multiple linear regression analysis indicated that the OTR and PR-B staining levels in ectopic endometrium were the only 2 co-variables that are associated with the depth of myometrial infiltration (OTR, positive association, *p* = 6.6×10^−15^, PR-B, negative association, and *p* = 0.0020, respectively; *R* ^*2*^ = 0.83).

### Effect of treatment on the number of GAD65-positive neurons in the brainstem nucleus raphe magnus (NRM)

**Fig. 8:**
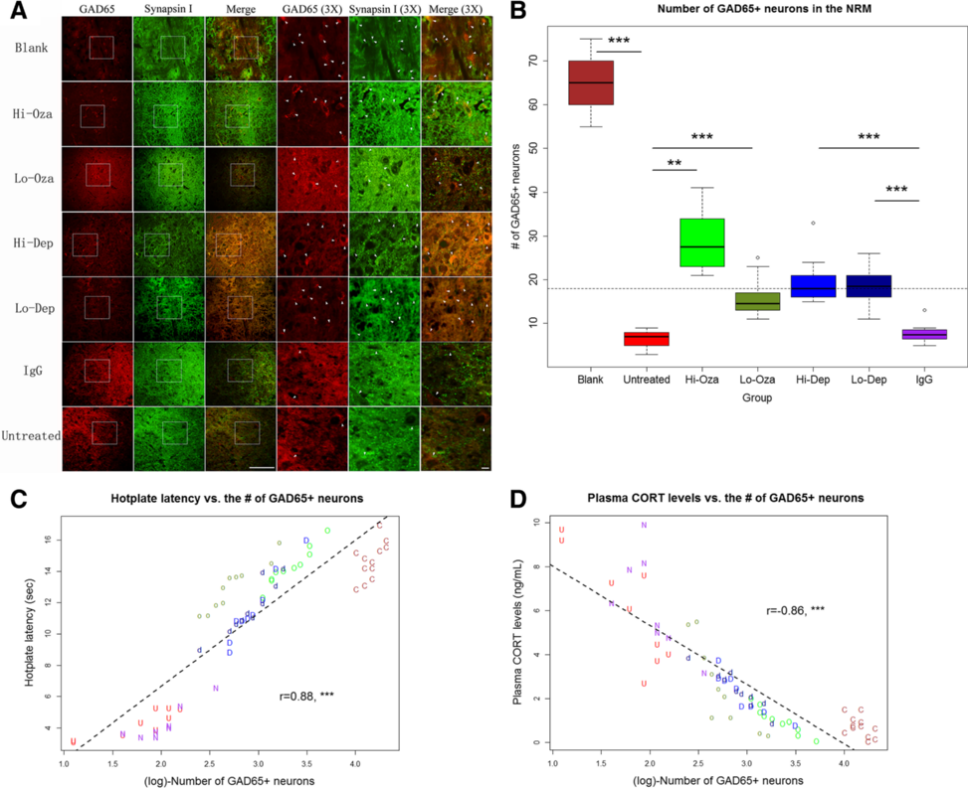
**a** Micrographs of immunofluorescent staining of GAD65 in the nucleus raphe magnus (NRM) in different groups of mice. Both GAD65- and Synapsin I-positive neurons were identified, as indicated by white arrows. To see the picture more closely, the area of interested was amplified three times. The original amplification:x400. The scale bar represents 125 μm. **b** Boxplot showing the number of GAD65+ neurons in the NRM among different treatment groups. The dashed line represents the median value of all mice. Blank: blank control endometrium; Hi-Oza: high-dose Ozagrel group; Lo-Oza: low-dose Ozagrel group; Hi-Dep: platelet depletion with high-dose antibody; Lo-Dep: platelet depletion with low-dose antibody; IgG: non-immune mock antibody. **c** Scatter plot of hotplate latency vs. the log-transformed number of GAD65-positive neurons in the NRM for all groups of mice; **d** Scatter plot of plasma corticosterone levels vs. the log-transformed number of GAD65-positive neurons in the NRM for all groups of mice. Each alphabet in the figure represents one experimental observation, and the alphabets are the abbreviations of different treatment groups. C: Blank control; U: Untreated; o: Low-dose Ozagrel; O: High-dose Ozagrel; d: Platelet depletion using low-dose antibody; D: Platelet depletion using high-dose antibody; N: Non-immune IgG. The correlation coefficient and its statistical significance levels are shown in (**c**) and (**d**). ***: *p* < 0.001

To see whether Ozagrel treatment and platelet depletion had any effect on the GABAergic inhibition system in the NRM, we performed an immunofluorescent staining of GAD65 in the NRM (Fig. 8a) and counted the number of GAD65-positive and synapsin I-positive neurons in the NRM. This number would be a measure of the number of GAD65-expressing neurons in the NRM.

We found that there is a significant difference in the number of GAD65-positive neurons in the NRM among the seven groups (*p* < 0.001; Fig. 8b). A multiple linear regression analysis (the number of cells was square-root transformed to improve normality) indicated that while adenomyosis induction was associated with the reduction in the number of GAD65-positive neurons in the NRM (*p* < 0.001) as previously reported [26] while both Ozagrel treatment and platelet depletion were associated dose-dependently with a significant increase of the number of GAD65-positive neurons (both *p*-values <0.001; *R* ^*2*^ = 0.92).

The number of GAD65-positive neurons (log-transformed) in the NRM was found to be positively correlated with the hotplate latency after treatment (*r* = 0.88, *p* < 0.001; Fig. 8c). It also was found to be negatively correlated with the plasma CORT levels (*r* = −0.86, *p* < 0.001; Fig. 8D).

### Factors associated with the uterine contractility

Both contractile amplitude and frequency correlated positively with the myometrial OTR staining levels (*r* = 0.82, *p* < 0.001, and *r* = 0.35, *p* < 0.01, respectively; Additional file 1: Figure S3A, B of Supplemental Information) and also with the lesional OTR staining levels in the ectopic endometrium (*r* = 0.94, *p* < 0.001, and *r* = 0.50, *p* < 0.001; Additional file 1: Figure S3 c, d of Supplemental Information).

For contractile amplitude, the multiple linear regression incorporating uterine weight/bodyweight ratio, depth of myometrial infiltration (0 if no adenomyosis), extent of platelet aggregation and macrophage infiltration and all immunostaining measurements in ectopic endometrium identified the depth of myometrial infiltration (*p* < 0.01), uterine weight/bodyweight ratio (*p* < 0.001), the extent of platelet aggregation (*p* < 0.001), and the number of infiltrating macrophages (*p* < 0.01) as four covariates that were associated with the contractile amplitude (*R* ^*2*^ = 0.94). For contractile frequency, we found, through multiple linear regression analysis, that only the uterine weight/bodyweight ratio (*p* < 0.001), lesional and myometrial OTR staining levels (both *p* < 0.05), and PR-B staining levels (*p* < 0.05) were associated with the contractile frequency (*R* ^*2*^ = 0.42).

### Determinants of thermal response latency after treatment

We carried out a multiple linear regression analysis to identify which factors potentially determine the change in thermal response latency before and after drug treatment using the pre-treatment latency, bodyweight, depth of myometrial infiltration (grade = 0 if no adenomyosis), uterine weight vs. bodyweight ratio, amplitude and frequency of uterine contraction, and presence of adenomyosis as covariates. We found that the uterine weight vs. bodyweight ratio (*p* < 0.01), contractile amplitude (*p* < 0.01), the presence of adenomyosis (*p* < 0.001) and the depth of myometrial infiltration (*p* < 0.001) were all negatively associate with the change in before-after hotplate latency (*R* ^*2*^ = 0.90).

## Discussion

We have provided evidence that anti-platelet treatment, through either platelet depletion or Ozagrel treatment, resulted in the suppression of myometrial infiltration, improved generalized hyperalgesia, reduced uterine weight vs. bodyweight ratio and stress level, and reduced amplitude and frequency of uterine contraction in mice with induced adenomyosis. The anti-platelet treatment also improved the expression of some proteins known to be involved in adenomyosis and reduced the number of infiltrating macrophages. In particular, it reduced the lesional expression of OTR, a SMM marker [29], and of collagen I and IV, markers of extracellular matrix deposits and thus fibrosis. Moreover, it increased the number of GAD65-expressing neurons in the brainstem NRM, thus likely boosting the GABAergic inhibition of pain due to adenomyosis, which in turn helps pain relief and reduces the stress level.

Our data are consistent with our finding that increased platelet aggregation and the extent of fibrosis in both mouse and human adenomyosis [10, 11]. They are also consistent with our previous report that anti-platelet therapy is effective in treating endometriosis in mouse [9, 14, 30] and that the expression of tissue factor in adenomyosis is elevated [5, 31]. Tissue factor plays a critical role in the initiation of platelet activation and coagulation [32]. In addition, considerable experimental data [33, 34] and limited clinical data [35] support the involvement of hyperprolactinemia in adenomyosis, yet prolactin is a potent cofactor for platelet aggregation [36, 37]. These data, taken together, seem to suggest that patients with adenomyosis may be in a hypercoagulable state as those with endometriosis [38]. This may explain the report of cerebral infarcts associated with adenomyosis [39] and increased mean platelet volume in women with adenomyosis [40].

Our data are also consistent with our previous reports that PR-B expression in adenomyosis is reduced [41] due possibly to PR-B promoter hypermethylation [42]. In addition, they are consistent with reported constitutive activation of NF-κB [5], increased expression of COX-2 [7], TRPV1 [43] and OTR in adenomyosis [29, 43, 44]. The increased uterine contractility, and, in particular, its close association with the OTR expression and with the reduced hotplate latency as reported in this study are consistent with what we reported in human adenomyosis [44]. In other words, the mouse model used in this study recapitulates several important features of human adenomyosis, i.e., inflammation and angiogenesis as displayed by the constitutive activation of NF-κB and increased COX-2 but decreased PR-B expression in adenomyotic lesions, increased uterine weight and presumably enlarged uterus, increased generalized hyperalgesia, and elevated uterine contractility, due possibly to elevated myometrial OTR expression. Remarkably, anti-platelet treatment either reversed or abrogated these changes.

We note that the anti-platelet treatment achieved the therapeutic effects very similar to EGCG [20, 26, 45] and resveratrol [46, 47] as we reported earlier. However, it is perhaps no coincidence that EGCG is anti-platelet [48] and so is resveratrol [46]. In fact, some compounds that are reported to be promising in treating adenomyosis in preclinical and clinical studies, such as andrographolide [2, 5, 49], valproic acid [19, 50, 51], and statins [52, 53], all turn out to be anti-platelet [54–57]. Even danazol, a once-popular, FDA-approved drug for treating endometriosis, has long been reported to have anti-platelet effect [58, 59].

That said, we should emphasize that, despite promising results of anti-platelet treatment by either platelet depletion or Ozagrel treatment as shown here, we are not advocating their use in clinical setting *per se*, even though Ozagrel is a prescription drug as of now. Adenomyosis is a benign disease and certainly not life-threatening. As such, it places higher premium on drug safety as compared with other life-threatening diseases such as cancer. While Ozagrel is generally safe and holds promises in treating adenomyosis, the hemorrhage risk it entails deserves caution. This study was meant to be a proof-of-concept study, demonstrating the therapeutic potential of anti-platelet therapy for adenomyosis. It is not intended to advocate Ozagrel *per se* for the treatment of human adenomyosis. More research is needed to determine which anti-platelet compound has the desirable benefit-to-risk ratio in treating adenomyosis.

While the exact mechanisms of action for anti-platelet therapy remain to be investigated, it is possible that anti-platelet treatment suppresses the activation of the TGF-β1/Smad3 signaling pathway and the expression of ER-β, both of which can be induced by activated platelets [15, 60]. In addition, the treatment suppresses the activation of NF-κB, which also can be induced by platelets (Zhang et al., unpublished data). Moreover, activated platelets express P-selectin (CD62P/GMP-140) on their cell surface [61–63], which binds to its ligand, P-selectin glycoprotein ligand-1 (PSGL-1), that is expressed on the cell surface of most leukocytes, such as neutrophils, monocytes, Th1 lymphocytes, eosinophils, and basophils, and facilitates inflammation, hemostasis, thrombosis, and the growth and metastasis of cancer [62, 64]. P-selectin interacts with PSGL-1, a transmembraine homodimer, to mediate the rolling of leukocytes on stimulated endothelial cells and the heterotypic aggregation of activated platelets and leukocytes [65], and activate mitogen-activated protein kinases (MAPKs) [66] and β_2_ integrins [67]. Hence, anti-platelet treatment should abrogate or attenuate inflammation caused by adenomyosis, as seen in reduced p-p65 expression in this study.

Anti-platelet therapy can also suppress neurite outgrowth and thus hyperinnervation in adenomyosis [68, 69] since ectopic endometrial stromal cells secrete platelet inducers such as thrombin and thromboxane A2 (TXA_2_) [70]. TXA_2_ has been reported to stimulate neurite outgrowth in cerebral cortical neurons [71], and we have also found that it can do so in dorsal ganglia root neurons [72]. Since TXA_2_, PGH_2_, and PGI_2_ have been reported to be potent inducers of uterine contractility [73] and uterine contractility is documented to be  correlated with the severity of dysmenorrhea in adenomyosis [44], the suppression of platelet activation and the resultant COX-2 down-regulation may suppress hyperinnervation and uterine hyperactivity, thus responsible for improved generalized hyperalgesia and reduced  plasma CORT levels.

The reduced plasma CORT levels in mice with anti-platelet treatment is likely to result from the modulation of chronic stress (adenomyosis-induced pain) response through GABA receptors as in a chick model of acute stress [74]. Alternatively, adenomyosis-induced pain or hyperalgesia may result in synaptic dysfunction, for example, HDAC-mediated impairment of GABA synaptic inhibition in the brainstem NRM [75]. However, whether suppression of platelet activation may restore the GABA synaptic inhibition in NRM through the reduction of HDAC activity remains to be clarified.

## Conclusions

This study further provides evidence that platelets play important roles in the development of adenomyosis. In addition, this study demonstrates that anti-platelet treatment is efficacious in suppressing myometrial infiltration, improving generalized hyperalgesia, reducing both uterine hyperactivity and systemic CORT levels in mice with induced adenomyosis. Collectively, these results demonstrate that anti-platelet therapy holds promises as a non-hormonal treatment for treating adenomyosis.

## Additional file

Additional file 1: Supplemental materials [76–80]. (PDF 358 kb)

## Abbreviations

CORT: Corticosterone
COX-2: Cyclooxygenase-2
EGCG: Epigallocatechin-3-gallate
EMT: Epithelial-mesenchymal transition
ER-β: Estrogen receptor β
FDA: FMT: fibroblast-to-myofibroblast transdifferentiation
GABA: *γ*-aminobutyric acid
GAD65: Glutamic acid decarboxylase 65
H&E: Hematoxylin and eosin
HD: Platelet depletion by high-dose rat anti-mouse GPIbα polyclonal IgG treatment
HDAC: Histone deacetylase
HO: High-dose Ozagrel treatment
LD: Platelet depletion by low-dose rat anti-mouse GPIbα polyclonal IgG treatment
LO: Low-dose Ozagrel treatment
MAPK: Mitogen-activated protein kinase
NI: Non-immune rat anti-mouse IgG isotope-matched with the anti-GPIbα antibody
NRM: Nucleus raphe magnus
OTR: Oxytocin receptor
PGH_2_: Prostaglandin H2, PGI_2_ prostaglandin I2
p-p65: Phosphorylated p65 subunit
PR-B: Progesterone receptor isoform B
PSGL-1: P-selectin glycoprotein ligand-1
ReTIAR: Repeated tissue injury and repair
SMM: Smooth muscle metaplasia
TGF-β1: Transforming growth factor β1
TRPV1: Transient receptor potential cation channel, subfamily V, member 1
TXA_2_: Thromboxane A2
