# Supplementary material for: Anti-platelet therapy holds promises in treating adenomyosis: experimental evidence
Source: Reprod Biol Endocrinol. 2016 Oct 10;14:66. doi: 10.1186/s12958-016-0198-1 (PMC5057470; doi:10.1186/s12958-016-0198-1)
Supplement: Additional file 1: — Supplemental materials [76–80]. (PDF 358 kb) [file 12958_2016_198_MOESM1_ESM.pdf]

Anti-Platelet Therapy Holds Promises in Treating Adenomyosis: Experimental Evidence

Bo Zhu, M. D.,Yumei Chen, M.D., Xiaolu Shen, M.D., Xishi Liu, M.D., Ph.D.,

Sun-Wei Guo, Ph.D.

Supplemental Information

Hotplate test procedures

The hotplate test is a commonly used method for measuring nociception in rodents [1]. It measures the response threshold to thermal stimuli, representing a form of acute thermal pain [2]. In this study, the hotplate test was performed with the Hot Plate Analgesia Meter (ZH-YLS-6B, Shanghai Beijing Industrial Company Ltd., Shanghai, China) consisting of a metal plate of 26×26 (cm) in size. The surface of the plate can be heated to a constant temperature of 55.0±0.1°C as measured by a built-in digital thermometer, and a plastic cylinder (22 cm in diameter, 12 cm in height) was placed on the hotplate. Mice were brought to the testing room and allowed to acclimatize for 10 minutes prior to the test. The latency to respond to thermal stimulus was defined to be the time (in seconds) elapsed from the moment when the mouse is inserted into the cylinder until it licks its hind paws or jumps. Each mouse was tested only once in each session. The latency was calculated as the mean of 3 readings recorded at intervals of 24 hours.

Measurement of uterine contractility

Uterine contractility was measured as described previously [3]. The right uterine of each mouse horn was dissected of deciduas and serosa in Ringer’s solution and then trimmed into uterine strips of approximately 2x2x10 mm in size, and then the strips were bathed in Kreb's solution consisting of 118 mM of NaCl, 25 mM of $NaHCO_3$, 1.2 mM of KH_2PO_4, 4.7 mM of KCl, 1.2 mM of $MgSO_4 \cdot 7H_2O$, 2.5 mM of $CaCl_2$, and 11.5 mM of glucose [4].

The contractile activity was recorded by ML845 PowerLab 4/25 Data Recording System (AD instruments, Sydney, Australia), as reported previously [5]. Briefly, uterine strips were incubated in an organ bath chamber (CW-3 Smooth Muscle Chamber, Shanghai Jide Experimental Apparatus Factory, Shanghai, China) filled

with 20 ml of Kreb's solution. The solution was maintained at 37°C and pH 7.4 at all time and was gassed continuously with a mixture of 95% O_2 and 5% CO_2. For measurement, one end of the uterine strip was tied to a Perspex holder and the other was tied under 1 g resting tension. All uterine strips were allowed to equilibrate for at least 1 hour during which the bathing solution was changed every 20 minutes. After the equilibration period, the spontaneous contractions were recorded for 10 min by Chart 5.0 software in the form of sinusoid-like wave as described by Calixto and Yunes, with a sampling rate of 2 Hz [6, 7]. Both the mean amplitude and mean frequency of the contraction were calculated for each mouse. The representative waveforms of uterine contraction in mice receiving different treatments are shown in Figure S1.

Measurement of plasma corticosterone level by enzyme linked immunosorbent assay (ELISA)

The corticosterone (CORT) ELISA Kit was purchased from Abcam (Hong Kong, China). The CORT measurement was performed following the manufacture's instruction, as reported previously [3]. Briefly, the harvested blood sample was collected in sterile tubes containing liquid EDTA, which were kept in cold ice. The samples were centrifuged at 2,000 g for 10 min and then the supernatant liquid (plasma) was harvested and stored at -20°C until use. The absorbance was read immediately on a microplate reader (Thermo Scientific Multiskan MK3, Waltham, MA, USA) at a wavelength of 450 nm. Then the mean optional density was converted into concentration. Each sample was evaluated in triplicate. The coefficient of variation was all less than 5%. The R^2 of the standard curve of the assay was greater than 0.98.

References

1. Le Bars D, Gozariu M, Cadden SW (2001) Animal models of nociception. Pharmacol Rev 53:597-652

2. Bannon AW, Malmberg AB (2007) Models of nociception: hot-plate, tail-flick, and formalin tests in rodents. Curr Protoc Neurosci Chapter 8:Unit 8 9

3. Chen Y, Zhu B, Zhang H, Liu X, Guo SW (2013) Epigallocatechin-3-Gallate Reduces Myometrial Infiltration, Uterine Hyperactivity, and Stress Levels and Alleviates Generalized Hyperalgesia in Mice Induced With Adenomyosis. Reprod Sci 20:1478-91.

4. Wang HW, Wu CC (2008) Effects of oxymetazoline on isolated rat's tracheal smooth muscle. Eur Arch Otorhinolaryngol 265:695-698

5. Mao X, Wang Y, Carter AV, Zhen X, Guo SW (2011) The retardation of myometrial infiltration, reduction of uterine contractility, and alleviation of generalized hyperalgesia in mice with induced adenomyosis by levo-tetrahydropalmatine (l-THP) and andrographolide. Reprod Sci 18:1025-1037

6. Calixto JB, Yunes RA (1991) Antagonism of kinin-induced contraction of isolated rat uterus by the crude hydroalcoholic extract from Mandevilla illustris. Gen Pharmacol 22:99-101

7. Hernandez-Magro PM, Villanueva Saenz E, Alvarez-Tostado Fernandez F, Luis Rocha Ramirez J, Valdes Ovalle M (2002) Endoanal sonography in the assessment of perianal endometriosis with external anal sphincter involvement. J Clin Ultrasound 30:245-248

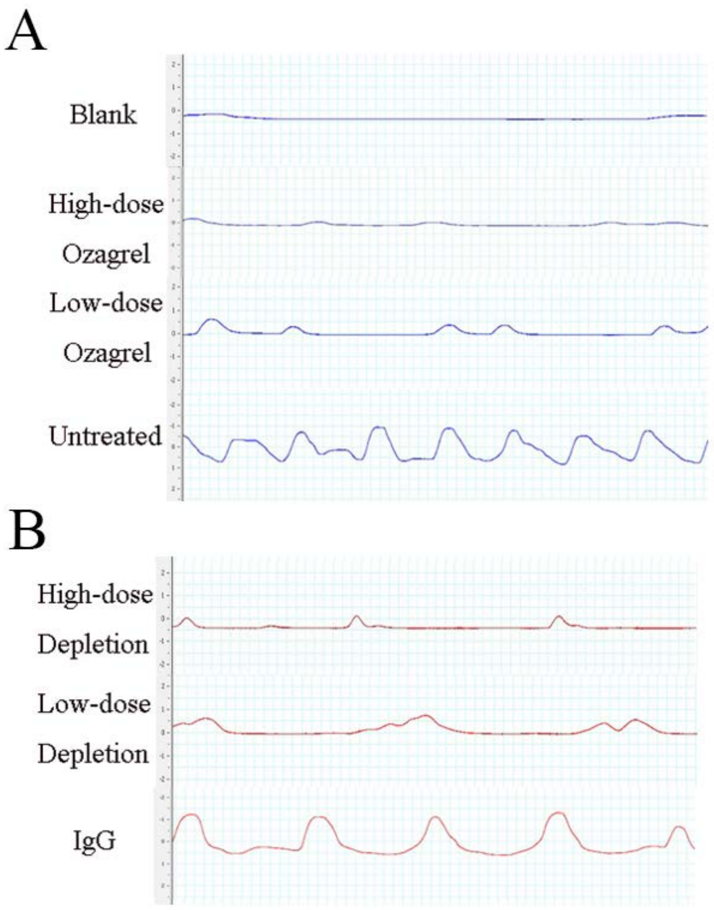

Figure S1. Representative waveforms of uterine contraction in mice receiving different treatments: (A) blank control group, high-dose Ozagrel group, low-dose Ozagrel group, and untreated group; and (B) high-dose platelet depletion group, low-dose platelet depletion group, and IgG group. One side of each small square represents 1 second in time.

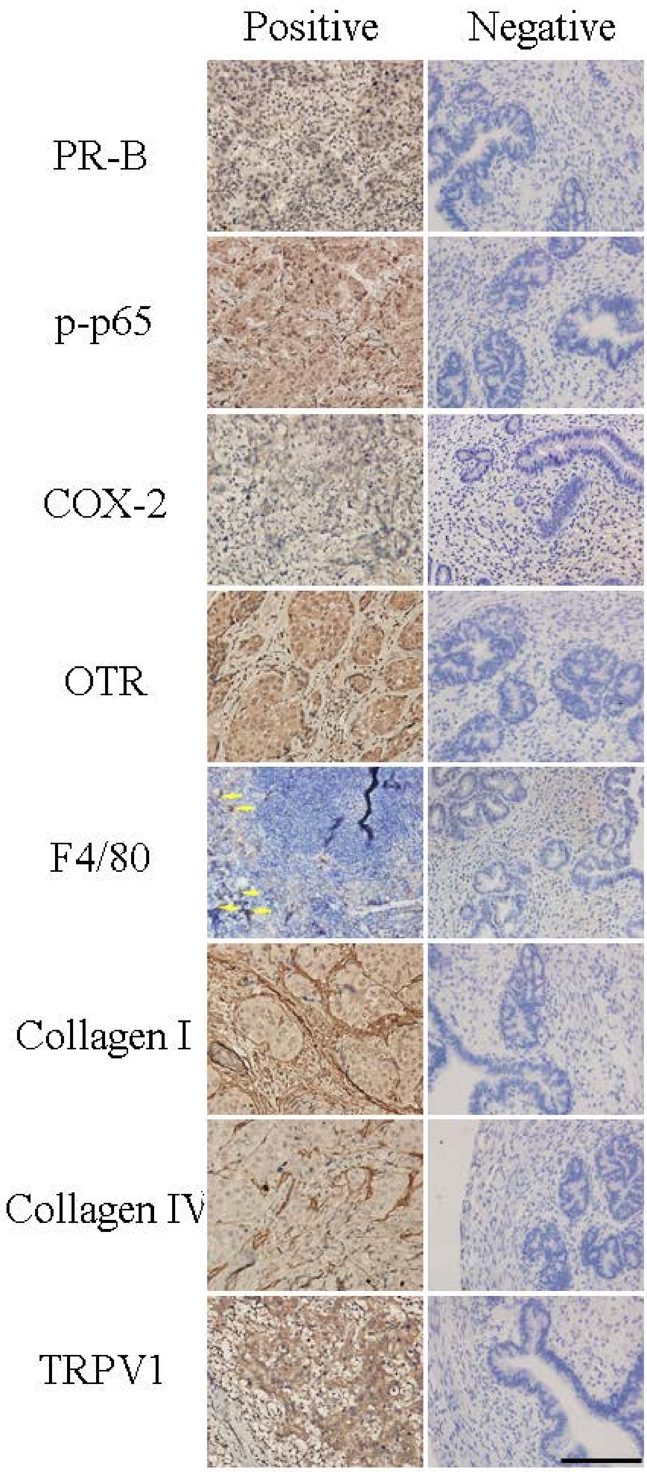

Figure S2. Positive and negative controls for immunostaining of PR-B, p-p65, COX-2, OTR, myometrial OTR, TRPV1, collagen I and IV, and the number of macrophages. Mouse spleen tissues were used for the positive immunostaining of macrophages, human breast cancer tissues, for the positive immunostaining of the other markers, while the sections for the negative immunostaining were from mouse's uterine tissues.

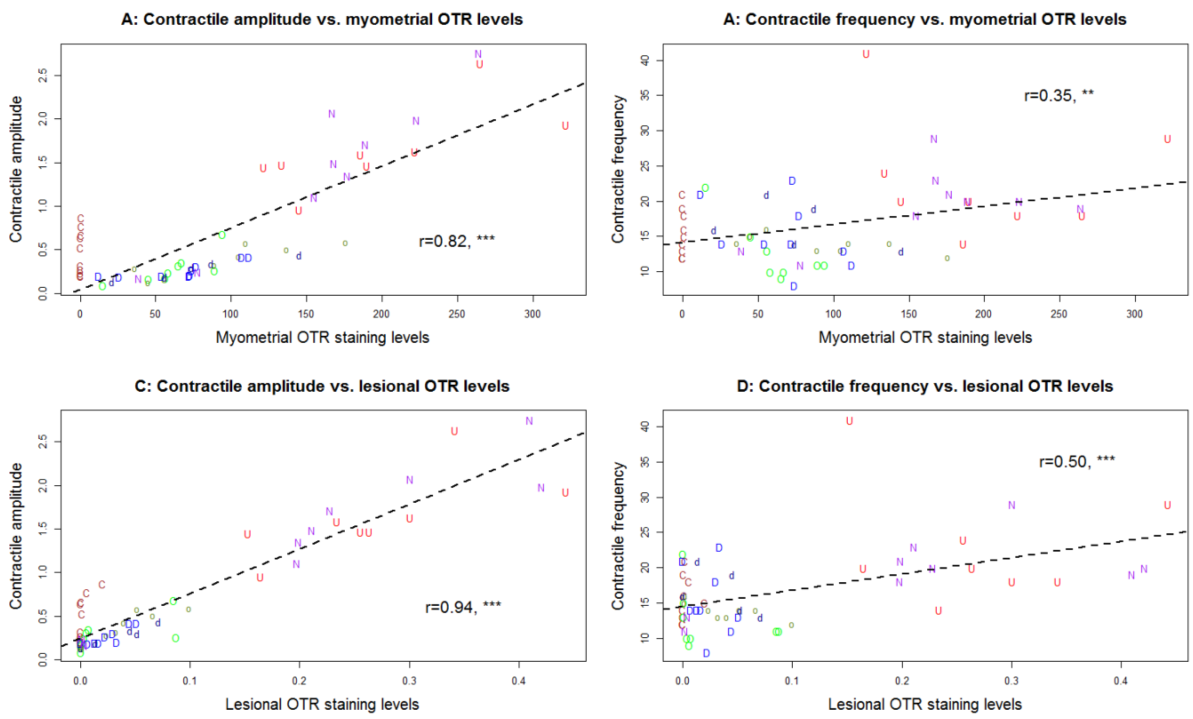

Figure S3. Scatter plots showing the relationship between the amplitude (A, C) and frequency (B, D) of uterine contraction and myometrial/lesional OTR staining levels. In all figures, each letter represents one mouse, and the alphabet indicates the group identity,. The correlation coefficient, with its statistical significance level, is shown in the figure. C: Blacnk control; U: Untreated; o: Low-dose Ozagrel; O: High-dose Ozagrel; d: Platelet depletion using low-dose antibody; D: Platelet depletion using high-dose antibody; N: Non-immune IgG. **: $p<0.01$; ***: $p<0.001$.
